# Cognitive vs. Linguistic Training in Children with Developmental Language Disorder: Exploring Their Effectiveness on Verbal Short-Term Memory and Verbal Working Memory

**DOI:** 10.3390/brainsci14060580

**Published:** 2024-06-05

**Authors:** Theodora Bachourou, Stavroula Stavrakaki, Vasiliki Koukoulioti, Ioanna Talli

**Affiliations:** 1 Center of Interdisciplinary Assessment, Counseling and Support (KE.D.A.S.Y.), 271 00 Ileia, Greece; dorabachourou@gmail.com; 2 Department of Italian Language and Literature, Aristotle University of Thessaloniki, 541 24 Thessaloniki, Greece; talli@itl.auth.gr; 3 Department of German Language and Literature, Aristotle University of Thessaloniki, 541 24 Thessaloniki, Greece; vasilikikou@del.auth.gr

**Keywords:** developmental language disorder, linguistic training, cognitive training, intervention effects

## Abstract

The present study explores comparatively the effectiveness of a cognitive (verbal short-term memory (vSTM), verbal working memory (vWM)) and of a linguistic training (10-week duration each) in the diffusion of gains in cognitive abilities (vSTM and vWM) of in school-aged Greek-speaking children with developmental language disorder (DLD). To this purpose, two computerized training programs i.e., a linguistic and a cognitive one, were developed and applied to three groups (A, B, and C) of children with DLD (N = 49, in total). There were three assessments with two vSTM tasks (non-word repetition and forward digit span) and a vWM task (backward digit span): pre-therapeutically (time 1), where no significant between-group differences were found, post-therapeutically I (time 2), and post-therapeutically II (time 3) and two training phases. In phase Ι, group A received meta-syntactic training, whereas group B vSTM/vWM training and group C received no training. In phase ΙΙ, a reversal of treatment was performed for groups A and B: group A received vSTM/vWM while group B meta-syntactic training. Again, group C received no training. Overall, the results indicated a significant performance improvement for the treatment groups and revealed beneficial far-transfer effects as language therapy can affect vSTM and vWM in addition to direct and near transfer effects. In addition, the intervention type order affected performance as follows: first, better performance on the vSTM task (non-word repetition) was shown when the linguistic treatment was delivered first; second, better performance on the vWM in Time 2 and Time 3 was shown by group B, for which the cognitive treatment was delivered first. Concluding, not only intervention type but also intervention type order can affect performance in DLD.

## 1. Introduction

Developmental language disorder (DLD) (previously known as specific language impairment) is defined as the difficulty with the acquisition of language skills, in all or some linguistic domains, including phonology, grammar, syntax, vocabulary, and pragmatics [1]. DLD affects about 8% of children [2], a number that undoubtedly requires the efficient implementation of clinical and evidence-based studies.

Among the biological and environmental etiology factors of the disorder that are now considered, family history, socio-economic status, or peri-natal problems should also be taken into account as inclusionary criteria for DLD. In addition, the new DLD inclusionary criteria set by the CATALISE group put an end to the obligatory differentiation between verbal and non-verbal intelligence and accept the coexistence of DLD with other neurodevelopmental disorders, such as ADHD, speech sound disorder, behavioral disorders, or below average non-verbal ability [3]. Children with DLD demonstrate mostly difficulties in morphosyntax [4,5,6], in non-canonical word order [7], and in the comprehension or/and production of relative clauses [8,9,10,11,12,13,14,15]. Moreover, children with DLD have difficulties in cognitive domains including phonological short-term memory [16], visuospatial working memory [17], executive function [18,19,20,21], cognitive flexibility [22], and processing skills [6,23].

Intensive research in the past was performed to identify whether the core clinical marker of children DLD concerned a deficit in linguistic or cognitive abilities. Many studies revealed that the core deficit in DLD is manifested in the domain of inflectional morphology, especially past tense marking in English-speaking children [24] or in the domain of complex syntax, especially in sentences formed though the operations of A-movement or A-bar movement [8,25,26,27]. On the other hand, other studies suggested that deficient performance on non-word repetition, a proper measure for vSTM, constitutes a strong index for DLD [28] or provided evidence that the core deficit of the disorder is not only linguistic but also cognitive (e.g., Plym et al. [29]). More recent studies underline the coexistence of cognitive and linguistic impairment in DLD and investigate their relation. For example, Gilliam et al. [30] have suggested that syntactic comprehension in children with and without DLD is strongly linked to four cognitive processes: fluid reasoning, controlled attention, complex working memory, and language knowledge in long-term memory.

Despite controversy, the above evidence is still useful for intervention schemes. Specifically, more efficient remediation of the deficits could be planned and applied if research findings are taken into account. It should be mentioned that so far a large body of intervention studies target the language deficits in DLD and not the cognitive or processing deficits [31,32,33]. However, recent studies provide encouraging results for clinicians needing to target multiple domains (both language and cognitive) in their intervention methods [34,35,36].

The main aim of the present study is to explore the effectiveness of cognitive targeted therapy and linguistically targeted therapy on the improvement of verbal short-term memory (vSTM) and verbal working memory (vWM) of individuals with DLD. While there is much evidence indicating that cognitive intervention targeting STM and/or WM contributes to enhancing these abilities in individuals with language disorders, there are only a few studies that explore far-transfer effects, namely the impact of linguistic intervention on vSTM and vWM.

### 1.1. The Effects of Cognitive Intervention on STM and WM

Several studies with children with impaired WM skills showed improvement in WM and academic performance after WM training [37,38,39]. Wener and Archibald [40] in a preliminary intervention study with two therapy schemes targeting language and WM skills in nine 7- to 9-year-old children (three with DLD, two with WM impairment, four with both language and WM impairment) revealed a domain-specific effect: children with WM impairment improved their WM skills, while children with DLD improved their grammatical skills and children with both language and WM impairment showed more gains in their grammatical than WM skills. There were also cross-domain effects for some children. The authors suggest that intervention is more effective when targeting children’s specific areas of weaknesses, which is why each child’s underlying deficit(s) should be identified before designing the therapy program. Pauls and Archibald [41] used a single-subject design to explore near-transfer (WM) and far-transfer (language, reading, math) effects of a WM training program [42] in a group of seven children (8–11 years old) with WM impairments (three of them with additional language impairments). They found immediate and long-term (3-month follow-up) near-transfer effects in all participants but modest far-transfer effects in four of the participants. None of the children with additional language impairments showed an improvement in language skills, suggesting that WM training does not generally lead to improvement in language skills and is not effective for children with language impairments. They conclude that participant-specific characteristics, such as age, training intensity, and baseline abilities of WM, which might influence transfer response, should be taken into account [41]. Furthermore, Henry et al. [43] applied a working-memory intervention for a group of children with DLD and explored its impact on the WM and language abilities of this group compared to an active control group that did not participate in training. They found that the group (therapy group vs. active control group) was a significant predictor for gains in WM skills and language intervention, indicating that a WM intervention can impact on untrained skills.

### 1.2. The Effects of Linguistic Intervention on STM and WM

While there are several intervention studies targeting remediating impaired linguistic and meta-linguistic mechanisms (e.g., see reviews by Cleave et al. [44] and systematic reviews by Cirrin and Gillam [45], Law et al. [46], Rinaldi et al. [47], Storkel et al. [48], for bilingual children see Nair et al. [49], Bettelli et al. [50]), only a few studies with a linguistic intervention scheme have explored the effect of linguistic therapy on non-linguistic abilities in DLD. In particular, Pauls and Archibald [36] provided a narrative-based language intervention (three 40-min individual sessions per week for five weeks) and investigated its impact on linguistic abilities, related academic abilities (reading and math), and working memory. The results indicated heterogeneity in improvement of linguistic or working memory abilities of these individuals. Moreover, two phonological awareness language interventions found improvement in verbal STM and WM skills [51,52] (first study: four 20-min individual sessions per week for four weeks, second study: two 15-min sessions per week in small groups for two semesters). These interventions included phoneme awareness and rhyming activities. By contrast, Swanson et al. [53] reported no effects of a narrative-based language intervention on verbal STM (three 50-min individual sessions per week for six weeks). Their goal was to increase the frequency of use of complex grammatical forms (subordinating conjunctions, coordinating conjunctions, complex verbs, postmodification of nouns) through narration of novel stories. Other studies failed to show far-transfer effects of language intervention (three 60-min individual sessions per week for five weeks) to WM skills [54] or found modest far-transfer effects on WM skills, i.e., in some children only [55]. However, none of the abovementioned studies report pilot applications of their experimental materials. Despite controversial findings, it has been proposed that clinicians can improve cognitive abilities, including WM, by teaching strategies for comprehending and producing language skills [7]. The idea behind this is that therapeutic schemas incorporate and activate cognitive mechanisms, so that linguistic knowledge learning becomes meta-cognitive.

The present study builds on previous research and further investigates the linguistic and cognitive intervention effects on cognition abilities (vWM and vSTM abilities) in individuals with DLD, by providing assessment of these abilities before (at time 1: T1) and after (at times 2 and 3: T2 and T3) two therapy phases (phase I and phase II). It innovates by applying two training programs for children with DLD, one linguistic/meta-linguistic and one cognitive, being undertaken one after the other. The linguistic/meta-linguistic training program focused on syntax and on complex syntactic structures, while the cognitive training program focused on vWM and vSTM skills. In this study, computerized therapeutic materials specifically designed for Greek-speaking individuals were employed. To the best of our knowledge this is the first study in Greek employing this particular design. An experienced speech and language therapist (the first author) administered individually the two 10-week training programs, with frequency of one session per week and duration of 40 min each session (i.e., a total of 6 h and 40 min of intervention). Three groups participated, two of them were the experimental treatment groups while the third one was the control group. In particular, the experimental clinical groups received both linguistic and cognitive treatment, successively, with different order. In the first therapy phase (phase I), while the clinical group A was receiving linguistic therapy (language-first group), the clinical group B was receiving cognitive therapy (cognitive-first group). In the second therapy phase (phase II), while the clinical group A was receiving cognitive therapy, the clinical group B was receiving linguistic therapy at the same time (T2). In sum, the present study combines both linguistic and cognitive training in children with DLD and explores direct and near-transfer as well as far-transfer effects in the domain of vSTM and vWM. Furthermore, it examines the effectiveness of the order of training pattern and applies the reversal of training schemes, combining both linguistic and cognitive training.

## 2. Materials and Methods

This study was performed within the PhD project of the first author (T.B.) under the supervision of the second author (S.S.) in accordance with the rules and regulations of Aristotle University of Thessaloniki. Within this PhD project, the effectiveness of cognitive and linguistic therapy on the linguistic and cognitive abilities of children with DLD was investigated. The present study focuses on reporting the linguistic vs. cognitive intervention effectiveness on cognitive abilities.

### 2.1. Participants

These children (mean age: 9;8 years, age range 7;3–12;0) had been diagnosed by the official governmental agency in Peloponnese for diagnosing learning and speech language difficulties in Greece (Centers of Interdisciplinary Assessment, Counseling and Support, KE.D.A.S.Y.) as having a severe language impairment by multidisciplinary groups of experts, including a psychologist who performed standardized tests (WISC-III [56]), a speech–language therapist for language assessment, a teacher of special education needs for literacy assessment, and a social worker for case history. The social worker interviewed parents to collect information about the child and his/her family. More specifically, information was collected about the developmental and medical history of the child (developmental milestones, signs of communication difficulties at preschool age, hearing problems, other medical diagnoses, etc.), as well as if there were language disorders in the family (i.e., relatives with a history of speech, language, or hearing difficulties), behavioral and school history, and the family’s socio-economic status. All children were monolingual, native speakers of Greek.

To assess their verbal and non-verbal IQ, the WISC-III was employed which has been standardized for Greek [56] and used in official diagnostic centers in Greece. The Greek version has been standardized for children 6 years–16 years 11 months. It consisted of two scales, non-verbal and verbal. The former includes the following subtests: picture completion, coding, picture arrangement, block design, object assembly, symbol search, and mazes; the latter includes the following subtests: vocabulary, similarities, arithmetic, information, comprehension, and digit span. The overall non-verbal IQ score for the participants with DLD was within the normal range (non-verbal IQ: >85). With respect to their verbal IQ, all participants of this study were assessed by means of digit span. They were required to repeat digits forward and backwards. The forward and backward digit repetition is considered a measure of verbal short-term memory and working memory, respectively. As described below (Section 2.2.1), these tasks were employed pre-therapeutically and post-therapeutically. All participants had a mental age at least 6 months below their chronological age in the digit span subscale of WISC-III^GR^ [56]. This performance is indicative of difficulties in the domain of short-term and working memory. All children attended mainstream classes. Furthermore, no children with pronounced hearing problems participated in the study. The children were diagnosed as having severe language problems. In addition, according to the official reports of clinicians, these individuals with DLD showed impairments in the domain of syntax as indicated by the group performances on a syntactic comprehension task (also employed in the study by Talli and Stavrakaki [15]). This test consisted of 36 sentences with relative clauses, passives sentences, and sentences with reflexive verbs. Accuracy was calculated as a percentage of correct responses to the sentences. The DLD group showed deficient performance compared to typically developing children of this age [15] as the scores obtained were below the average score of typically developing children of this age. Additional difficulties in language for these children were detected by means of the Reading Test A [57]. This is a standardized diagnostic tool that has been developed to assess reading abilities in Greek-speaking children aged 8–15 years [57]. It evaluates four subdomains: decoding, fluency, morphosyntax, and reading comprehension. In the morphosyntax subscale, expressive morphosyntactic skills are assessed as follows: (a) production of verbs (person, number, tense, and aspect marking), (b) production of morphologically complex words, (c) sentence elicitation with visual cue, and (d) sentence elicitation without visual cue. Scores are converted into percentiles, which are the normative data for this test (50th percentile is the mean score). All children of this study had a percentile of 30 or below in morphosyntax, which is 2 SD below the mean. Difficulties in the domain of morphosyntax indicate the presence of a language disorder in the participants of our study.

In addition, all participants performed non-word repetition [58,59] in which they had a percentile below 1. This task assesses verbal STM. It is a Greek adaptation from the French test battery EVALEC [60] (Greek adaptation: [58]; see [59] for normalization for Greek children and adolescents aged 7–13 years old). It includes twenty-four three- to six-syllable non-words (six non-words for each length: four with CV and two with CVC syllable structure) presented orally in increasing length order. The child had to repeat each non-word after the examiner and his/her responses were noted. Accuracy score was the total number of correctly repeated syllables, calculated as a percentage. The children had a percentile below 1 in the task, which indicates deficient performance in vSTM. This performance characterizes individuals with DLD [16].

All participants were randomly assigned to three groups: (1) group A (linguistic-first group) consisted of 16 children with DLD receiving meta-linguistic training first and then vSTM/vWM training; (2) group B (cognitive-first group) consisted of 17 children with DLD receiving vSTM/vWM training first and then meta-linguistic training; (3) group C (control group) consisted of 16 children with DLD who received only their usual training at school (support by special education teachers). Notably, all children followed their usual everyday schedule. They were receiving special education services, either at their public school (inclusion class) or at home after school with a private teacher. While the effect of these factors cannot be evaluated, it is underlined that they were in action for all the participants of this study. In Table 1, demographic and clinical characteristics of these groups are presented. The groups did not differ in chronological age and non-verbal IQ. With respect to chronological age (CA), a Kruskal–Wallis test for non-parametric data was performed, as the distribution of the cognitive first group was not normal, which showed no group effect (*x*^2^(2) = 2.7, *p* = 0.3). With respect to the non-verbal IQ we performed a Kruskal–Wallis test for non-parametric data, as the distribution of the language-first group was not normal, and again the difference was not significant (*x*^2^(2) = 1.1, *p* = 0.6). For syntactic comprehension, we performed a one-way ANOVA, which showed that the three groups did not differ from each other (*F*(2, 46) = 0.76, *p* = 0.47).

### 2.2. Assessment Material and Procedure

#### 2.2.1. Pre- and Post-Training Assessment Materials

There were three assessments: one pre-therapeutically and two post-therapeutically (one week after the first training and one week after the second training). This paper focuses on assessments performed by means of two verbal STM tasks and one verbal WM task. Additional detailed linguistic testing was also performed [61]. Assessments were performed by the first author (T.B.), an experienced speech and language therapist.

##### Verbal STM

Non-word repetition: this task assesses verbal STM. As described above (in Section 2.1), this task assesses vSTM by non-word repetition (twenty-four three- to six-syllable non-words (six non-words for each length: four with CV and two with CVC syllable structure). The task is presented orally in increasing length order. As pointed out above, the accuracy score was the total number of correctly repeated syllables (%).

Forward digit span: this task also assesses verbal STM. It is the subtest of the Greek WISC-III [56]. The child was asked to repeat a list of digits (2–9 digits) in increasing order which were presented orally by the examiner. It consists of eight lists, for each of which the child could complete two trials: every correct trial was awarded with 1 point. The interruption criterion was a score of zero in both trials of a list. Total raw scores were taken into consideration (maximum 16 points).

##### Verbal WM

Backward digit span: this task assesses verbal working memory and is also the subtest from the Greek WISC-III. It consists of seven lists presented in increasing length (2–8 digits) and the child had to repeat them backwards. For each list the child could complete two trials: for a correct response from the first trial s/he was awarded with 2 points and for a correct response in second trial 1 point. When the child failed in both trials of a list the administration would stop. Total raw scores were noted (maximum 14 points).

#### 2.2.2. Intervention Material

##### Training Programs

**Figure A1:**
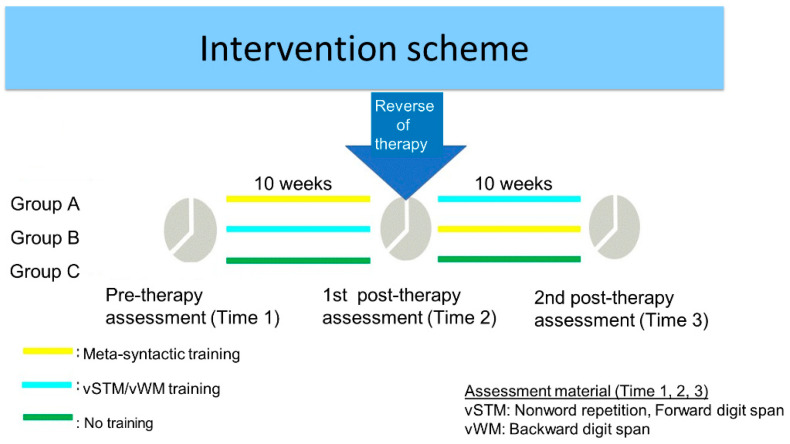
Overview of the intervention scheme, its duration and assessment material.

There were two training phases for both groups A and B. Group C received no training. Each of the two interventions included a 10-week training program once a week (40 min each session) for each child with DLD that was delivered individually. Intervention materials were administered by means of two software applications for a tablet. Intervention was applied by the first author (TB), an experienced speech and language therapist. In phase Ι, group A received meta-syntactic training, whereas group B vSTM/vWM training. In phase ΙΙ, a reversal of training was performed for groups A and B: group A received vSTM/vWM while group B meta-syntactic training. In Figure A1 (Appendix A), the intervention phases are schematically presented. The intervention design developed in this study, in line with other studies in the field [34,62], aimed to evaluate the direct and near-transfer as well as far-transfer effects of linguistic vs. cognitive intervention in the domain of vSTM and vWM, in addition to the effect of the order of training pattern. For each post-therapy time (T2 and T3), the short-term goals for the groups that received intervention concerned the improvement of vSTM and vWM abilities via application of different training patterns. By the reversal of the therapy scheme order, the direct/near-transfer vs. far-transfer effects can be evaluated. Assessment was performed one week after the completion of each therapy pattern (after T2 and after T3) to evaluate the short-term goals. While our study, in line with other intervention studies [34,62], was not designed to provide a follow-up to assess whether there are permanent beneficial effects on the vSTM and vWM abilities of individuals with DLD, there was a longer-term goal than the short-term one described above. This was to evaluate the effect of the overall therapy scheme by assessing whether the therapy type provided first (linguistic vs. cognitive) after T1 affected the performances shown in T3. By so doing, our long-term aim was to assess the effect of the intervention type order on performances in T3.

##### Meta-Syntactic Training

Meta-syntactic training was delivered through a software application named Assisted Language Acquisition (AS.L.A. URL: https://lab7.gr/asla.html [63]) comprising both production and comprehension of relative clauses. The theoretical background for the development of the tasks was, on the one hand, tests developed in the context of complex structures in DLD [10,25] and, on the other hand, therapeutic approaches with a theoretical background, see Ebbels [33] for an overview. There was a pilot application of the software by an experienced speech and language therapist to five children with DLD (grades 2–5) for at least 4 sessions, in order to check the acceptance and applicability of the software to this population. The software that was developed, exclusively for research and academic purposes, can run both on Windows operating systems and on tablets and/or mobile devices running the Android operating system. There is also the possibility of future transfer to other platforms, such as iOS. Among the various application development solutions available, the RAD Studio XE65 application development environment from Embarcadero was chosen (URL: https://www.embarcadero.com/products/rad-studio) and, in particular, the free trial version of the environment., Notably, all children had previous experience with tablets and they were familiar with their use.

A combination of linguistic and meta-linguistic techniques was used for the training of relative clauses, both production and comprehension. For the comprehension of relative clauses, at first the child would listen to a sentence with relative clauses and then s/he had to represent the sentence visually, through a specially configured interface. Visual representation through pictures [64,65] and symbols (see [33] for an overview) helps the child to understand the knowledge of how the object relative clause is connected and how it specifies the object of the main clause. The construction of the sentence was performed by choosing the correct image among others for every term of the sentence (i.e., subject, verb, object, subordinate relative clause). Different shapes and colors were chosen (see Ebbels [33]) for every term of the sentence: a subject was represented with a blue circle, a verb was represented with a green triangle, an object with a red square, a subordinate relative clause with a red diamond. A subordinate relative clause had the same color as the object to show the link between them. Visual representation with symbols and colors of the function and the relationship of the terms of the sentence aims at the activation of the conscious representation of the syntactic relation, that is, the meta-syntactic awareness.

See (1) for an example (stimulus sentence in Greek, in IPA, glossed and with translation in English):

(1)   Το    αγόρι     κοιτάζει τη γιαγιά που      αγκαλιάζει η   γυναίκα.
  to     aγori      citazi   ti       jaja       pu         agaʎazi        i     jineka.
  The boy_NOM_ looks the grandma  who_ACC_ hugs          the woman_NOM_
  ‘The boy looks at the grandmother, who the woman hugs’.

In case the child misrepresented the sentence, the clinician would use a recasting technique, s/he would repeat the sentence and would indicate to the child the correct representation of the sentence, again explaining to him/her how the object relative clause is connected with the main clause.

After training comprehension, the production of relative clauses was then trained through elicitation technique [25]. A script was presented to the child with two pictures who was asked to answer the clinician’s question, which contained the target structure of relative clauses, with partial guided production. See (2) for an example of production of subject relative clause (the introductory sentence, the prompting questions, and the guiding utterance of the clinician are in English, the target sentence is in Greek, IPA, glossed and translated into English).

(2)   **Introductory sentence:** 
  Here are two pictures with children. One child is cooking soup, the other child is eating ice cream.
  **Prompting question**
  Which child would you rather be?
  **Experimenter utterance for partial guiding:**
  I would rather be…
  **Target:**
  … το   παιδί         που   τρώει  παγωτό.
  … to   peδi            pu     troi      paγoto
  … the child_NOM_  who  eats     ice cream.
  … the child who eats ice cream.

Feedback was given by the clinician according to the correctness of the child’s answers. The children received a small prize at the end of each session (stickers or snack). The child’s score/performance was recorded by the clinician during the treatment and after the session from the application statistics. All sessions were recorded and could be extracted in a file for further examination.

##### vSTM/vWM Training

Verbal STM and WM training was delivered through an application named Boosting Memory (BOOM! [66]). Similarly to AS.L.A., BOOM! software (URL: https://lab7.gr/boom.html) was developed to run both on Windows operating systems and on tablets and/or mobile devices running the Android operating system. There is also the possibility of future transfer to other platforms, such as iOS. Training material was divided into 10 sessions of varying difficulty. Each session, delivered individually, included five different types of tasks, each focusing on an area of phonological short-term and working memory. The order that was followed in each session is as follows: a. digit span, b. backward digit span, c. word repetition, d. non-word repetition, e. sentence repetition, f. N-back task (recall of penultimate word of a sentence), g. N-back task (recall of last two words of a sentence). For the specific design, research data of clinical interventions for STM and WM training for individuals with language disorders have been taken into account (for intervention in aphasia, Salis et al. [67], Salis [68] and for intervention in children with DLD [35]). Similarly to AS.L.A., there was a pilot application of the software by an experienced speech and language therapist to five children with DLD (grades 2–5) for at least 4 sessions in order to check the acceptance and applicability of the software to this population. In the forward digit span the child must repeat aloud in the same order a series of digits increasing in length from 4 to 9, such as 4-7-2-1, 2-6-4-7-8-3-9-5-1. The level of difficulty increased from session to session. In the backward digit span the child must repeat aloud a series of digits increasing in length from 4 to 9 in the reversed order, such as 5-7-3-8, 8-3-7-5. Similarly to the forward digit span, the level of difficulty increased from session to session. In the word repetition task, the children had to repeat words increasing in length from one to five syllables, with the syllabic structure CV or CVC, e.g., mati (=eye), nero (=water), meli (=honey). In the non-word repetition task, children had to repeat non-words with increasing length from one to five syllables and syllabic structure CV or CVC, e.g., loma, taci, nati, tola, kapatoli. In the sentence repetition task, children had to repeat aloud a sentence. The length of sentences increased in number of words from session to session. See (3) and (4) for examples (sentences in Greek, in IPA, glossed and with translation in English).

(3)   O     κηπουρός κόβει ένα όμορφο   τριαντάφυλλο.
  O     cipuros      kovi   ena omorfo     triantafilo.
  The  gardener   cuts   a      beautiful  rose.
  ‘The gardener cuts a beautiful rose’.

(4)   O     κηπουρός ποτίζει  τον   όμορφο    μεγάλο κήπο.
  O     cipuros     potizi      ton   omorfo     meγalo  cipo.
  The gardener   waters    the    beautiful  large      garden’.
  ‘The gardener waters the beautiful large garden’.

For the N-back tasks children had to recall the penultimate and the last two words of a sentence, respectively. See (5) for an example of the task of recall of the penultimate word of a sentence (sentences in Greek, in IPA, glossed and with translation in English, the target word in bold):

(5)   H     γυναίκα ζωγραφίζει τον **μεγάλο** καμβά
  I       jineka      zoγrafizi      ton  **meγalo**  kamva.
  The  woman   paints           the  **large**      canvas.
  ‘The woman paints the large canvas’.

In each session the child follows a story script presented through graphics reminiscent of cartoons. The story goes like this: on a beach there is a treasure chest. The child listens to recorded instructions at each stage of the session, as well as sentences, words, etc., which s/he must repeat in the same order. As the examination progresses, the chest seems to open every now and then. A pirate appears at the right moment, whose purpose is to take the treasure. Depending on the child’s performance and the answers s/he gives as the process progresses, the pirate will eventually open the chest and find either the treasure or a pumpkin inside. Depending on the child’s answers the clinician touches the screen to the right for correct answer or to the left for wrong answer. Feedback was given by the clinician after each answer according to the correctness of the child’s answers. The children received a small prize at the end of each session (stickers or snack).

## 3. Results

The group performances on the assessment measures in T1, T2, and T3 are presented in Table 2.

### 3.1. Data Analysis

We performed three types of analyses. First, the data from the non-word repetition task were analyzed by means of multilevel models for mixed designs, since the design included one within-subject (testing time) and one between-subject (group) factor. For the factor group we used sliding contrasts, which means that the two treatment groups were compared to each other and the cognitive-first group was compared to the control group. For the factor testing time we set sliding contrasts, too, so that each level was compared to its adjacent level, i.e., testing time 1 was compared to testing time 2 and testing time 2 was compared to testing time 3. For these analyses we also included the following random components: an adjustment of each child’s individual average (random intercept for subjects) and an adjustment for each child’s testing time effect (random slope for subjects). For each analysis we followed the following procedure for choosing the best model. First, we fitted a model with only the intercept and subsequently added the main effects testing time and group and finally the interaction of the two factors. Then, we compared the models and if there was a significant difference between two models, it was considered as a significant contribution of the additional factor. Finally, we used the contrasts in order to break down the interactions.

The second type of analysis consisted of one-way repeated measures ANOVAs and concerned within-group comparisons across testing times, whereby we defined sliding contrasts for the factor testing time so that each level was compared to its adjacent level, i.e., testing time 1 was compared to testing time 2 and testing time 2 was compared to testing time 3, as in the multilevel model. Similarly, we fitted models only with the intercept and then added the main effect. A significant difference between the models was considered as a significant effect of time. These analyses were necessary because the multilevel models for mixed designs provide information about the difference between groups concerning the change from testing point to testing point and not the performance of each group across testing times. We also performed one-way ANOVAs for between-group comparisons at different testing times. We defined sliding contrasts for the factor group, so that the language-first group is compared to the cognitive-first group and the cognitive-first group is compared to the control group. The interpretation was the same as for the repeated measures ANOVA.

The third type of analysis concerned the data from the forward and backward digit span, which were analyzed by means of non-parametric tests because the distributions were not normal and no transformation could be applied to overcome this problem. In particular, we performed a non-parametric Kruskal–Wallis test for the between-group comparisons for each testing time. For the post hoc comparisons, we used the Dunn test, which is the most popular for post hoc comparisons after a significant Kruskal–Wallis, with Benjamini–Hochberg corrections to control the familywise error rate. For the pairwise comparisons we report the adjusted *p*-value. For the within-group analysis for each testing time we used the non-parametric Friedmans’s chi-square test, which corresponds to the one-way ANOVA for repeated measures, and performed post hoc comparisons with the Wilcoxon test for repeated measures by adjusting the *p*-values with Bonferroni correction. All analyses were carried out with the R software [69].

#### 3.1.1. Verbal Short-Term Memory—Non-Word Repetition Task

**Figure 1:**
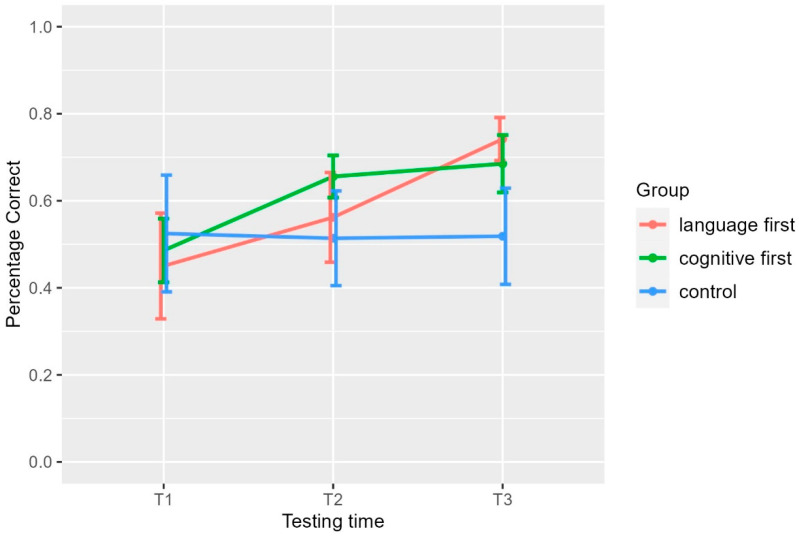
Percentage of the correct responses in the Verbal Short Term Memory (Non-Word Repetition Task). T1: pre-training assessment, T2: 1st post-training assessment, T3: 2nd post-training assessment, language first: group which received first meta-linguistic training and second STM/WM training, cognitive first: group which received first STM/WM training and then meta-linguistic training, control: control group which received no training.

Figure 1 presents the percentage correct responses of the verbal short term memory task (non-word repetition) for the three groups at the different testing times; see also Table 2.

The one-way ANOVA for T1 showed no group effect in the pre-treatment time (*β* = 3.85, *t* = 0.91, *p* = 0.37 for the comparison between language first and control and *β* = 4.22, *t* = 0.1, *p* = 0.32 for the comparison of cognitive first and control). The comparison of the different models for the whole dataset revealed significant main effects of group (*x*^2^(6) = 10, *p* = 0.0077) and testing time (*x*^2^(8) = 63, *p* < 0.0001). Moreover, we found a significant interaction of group by testing time (*x*^2^(12) = 93, *p* < 0.0001). We used the contrasts to gain insight into this interaction. Table 3 shows the β, the *t*-value, the *p*-value, and the effect size for each contrast.

The results indicate that non-word repetition improved for both treatment groups. In particular, the cognitive-first group improved by T2, i.e., after receiving the cognitive intervention, in comparison to both the language-first group (but the effect size is medium) and the control group (large effect). Concerning the change between T2 and T3, the language-first group improved significantly more than the cognitive-first group with the effect being large, whereas the cognitive-first group did not improve further after receiving the meta-linguistic intervention, i.e., it remained as stable as the control group. The one-way ANOVA for T2 showed that the cognitive-first group was significantly better than both the language-first (*β* = 10.14, *t* = 2.99, *p* = 0.0045) and the control group (*β* = −15.3, *t* = −4.5, *p* < 0.001). The post hoc *t*-test for the comparison between the control and the language-first group showed that there was no difference between them (*t*(29.9) = 1.28, *p* = 0.63) Interestingly, the one-way ANOVA for T3 showed that the language-first group was significantly better than the cognitive-first group (*β* = −6.13, *t* = −2, *p* = 0.046), which was better than the control (*β* = −18, *t* = −6, *p* < 0.0001). This finding suggests that the group that received the meta-linguistic intervention first not only improved in non-word repetition but also achieved higher performance in T3 than the treatment group that first received the meta-cognitive and afterwards the meta-linguistic intervention. The within-group comparisons showed that the language-first group improved significantly both from T1 to T2 (*β* = 12, *t* = 5.4, *p* = 0, *r* = 0.7, large effect) and from T2 to T3 (*β* = 19, *t* = 8.6, *p* = 0, *r* = 0.8, large effect). Concerning the cognitive-first group, it improved only from T1 to T2 (*β* = 18, *t =* 10, *p* = 0, *r* = 0.87, large effect) and not from T2 to T3 (*β* = 3, *t* = 2, *p* = 0.1). The control group did not improve across time, as expected.

#### 3.1.2. Verbal Short-Term Memory—Forward Digit Span

**Figure 2:**
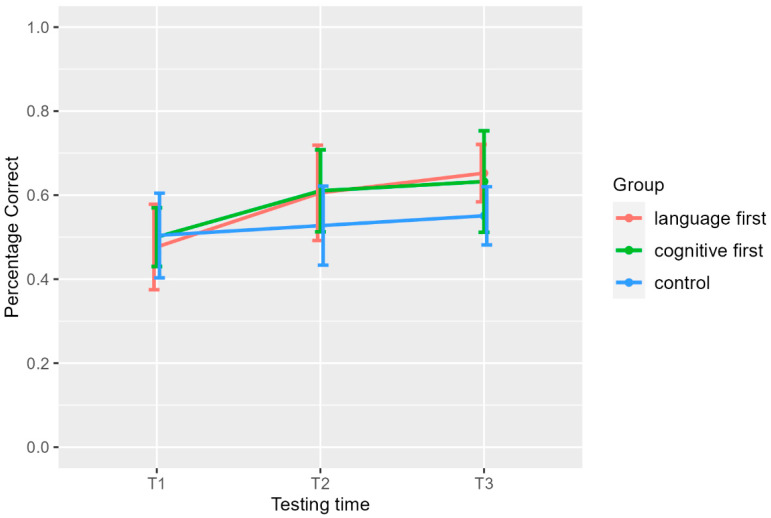
Percentage of correct responses in the Verbal Short-Term Memory (Forward Digit Span Task). T1: pre-training assessment, T2: 1st post-training assessment, T3: 2nd post-training assessment, language first: group which received first meta-linguistic training and second STM/WM training, cognitive first: group which received first STM/WM training and then meta-linguistic training, control: control group which received no training.

Figure 2 presents the percentage of correct performances in the forward digit span, the second measure of verbal STM, for exact numbers, see Table 2. As the figure shows, the two treatment groups manifest the same pattern. This was confirmed by the statistical analysis.

There was not a significant group effect in T1 (*x*^2^(2) = 0.9, *p* = 0.6), and the effect just failed to reach significance in T2 (*x*^2^(2) = 6, *p* = 0.06) but was significant in T3 (*x*^2^(2) = 10, *p* = 0.005). The post hoc tests for T2 showed non-significant differences between the two treatment groups and the control group, (language first vs. control: *Z* = −1.93, adjusted *p* = 0.08 and cognitive first vs. control: *Z* = 2.22, adjusted *p* = 0.08). The difference between the two treatment groups was not significant either (*Z* = 0.26, adjusted *p* = 0.8). Post hoc comparisons for T3 showed a significant difference between language first and control (*Z* = −3.14, adjusted *p* = 0.005), with the effect being large (*r* = 0.56, large), and between cognitive first and control (*Z* = 2.25, adjusted *p* = 0.037), but the effect was moderate (*r* = 0.35). As in T2, there was no difference between the two treatment groups (*Z* = −0.95, adjusted *p* = 0.34). Summing up the between-group comparisons, we see a difference for the two treatment groups compared to the controls only in T3 after both treatment groups received both interventions. We suggest that the moderate vs. large effect is due to the large vs. small standard deviation observed in T3 for the cognitive-first and the language-first groups, respectively, although they have very similar mean performance. Within-group comparisons yielded significant effects of testing time for the two treatment groups (for the language-first group, Friedman’s *x*^2^(2) = 21, *p* = 0 and for the cognitive-first group, Friedman’s *x*^2^(2) = 16, *p* = 0). Pairwise comparisons showed a significant difference between T1 and T2 (*W* = 2.5, adjusted *p* = 0.002, *r* = 0.86, large) but not between T2 and T3 (*W* = 24, adjusted *p* = 0.2, *r* = 0.46, moderate) (Bonferroni correction) for the language-first group. A similar pattern manifested for the cognitive-first group, namely a significant difference between T1 and T2 (*W* = 8, adjusted *p* = 0.006, *r* = 0.76, large) but not between T2 and T3 (*W* = 15.5, adjusted *p* = 1, *r* = 0.2, small). Surprisingly, the Friedman’s test was also significant for the control group (Friedman’s *x*^2^(2) = 8, *p* = 0.01). The post hoc pairwise comparisons between T1 and T2 and between T2 and T3 were not significant (*W* = 15.5, adjusted *p* = 0.68 and *W* = 9, adjusted *p* = 0.66, respectively). However, the difference between T1 and T3 was significant (*W* = 0, adjusted *p* = 0.02, *r* = 0.74, large). In sum, both treatment groups showed a large improvement in T2 compared to T1, whereas there was no substantial change between T2 and T3. Concerning the control group, there was an overall improvement from the T1 to T3.

#### 3.1.3. Verbal Working Memory—Backward Digit Span

**Figure 3:**
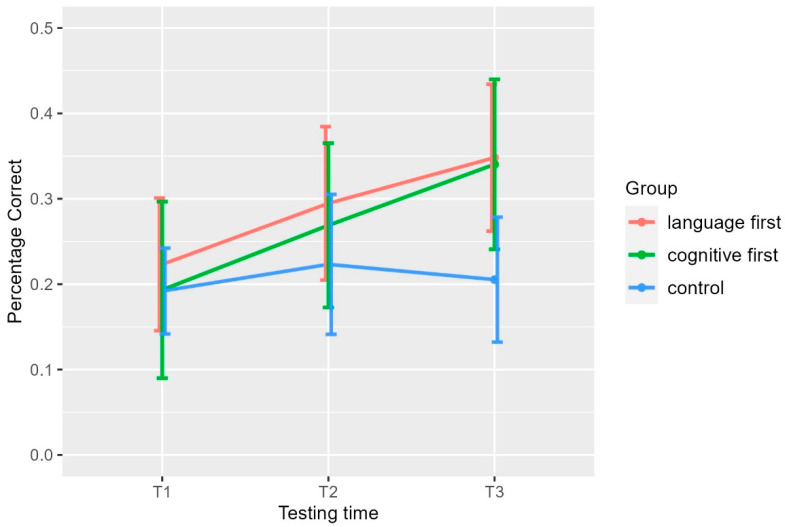
Percentage correct responses in the Verbal Working Memory (Backward Digit Span Task). T1: pre-training assessment, T2: 1st post-training assessment, T3: 2nd post-training assessment, language first: group which received first meta-linguistic training and second STM/WM training, cognitive first: group which received first STM/WM training and then meta-linguistic training, control: control group which received no training.

The percentage of correct performances in this task is shown in Figure 3 and Table 2. While the performance of both treatment groups is very low, as it remains at below 50% correct, significant improvement in the treatment group performance was found as shown by the pairwise comparisons presented below.

The between-group comparisons for each testing time showed no group effect for T1 (*x*^2^(2) = 1, *p* = 0.5) and T2 (*x*^2^(2) = 5, *p* = 0.09), whereas there was a difference in T3 (*x*^2^(2) = 19, *p* = 0). The post hoc tests for T3 showed that the difference between the two treatment groups and the control group was significant and the effect sizes were large for both comparisons (language first vs. control, *Z* = −3.9, adjusted *p* = 0, effect size *r* = 0.7 and cognitive first vs. control *Z* = 3.71, adjusted *p* = 0, *r* = 0.63), whereas the difference between the two treatment groups was not significant (*Z* = −0.25, adjusted *p* = 0.8). Summarizing, neither treatment resulted in between-treatment-group differences in BWDS. In T3, when both treatment groups had received both therapies, the treatment groups were significantly better than the control and the effect was large.

Similarly to the forward digit span task, we performed the non-parametric Friedman’s ANOVA for the within-group comparisons. For the language-first group there was a significant effect of testing time (Friedman’s *x*^2^(2) = 15, *p* = 0.0007). Pairwise comparisons showed a significant difference between T1 and T3 (*W* = 10, adjusted *p* = 0.025, *r* = 0.7, large effect), but not between T1 and T2 (W = 22, adjusted *p* = 0.171) and between T2 and T3 (*W* = 9, adjusted *p* = 0.1). This suggests a gradual improvement. There was also an effect of testing time for the cognitive-first group (Friedman’s *x*^2^(2) = 15, *p* = 0.0006). The pairwise comparisons showed that both the differences between T1 and T2 and between T2 and T3 were significant and the effect was large (for T1 vs. T2 *W* = 14, adjusted *p* = 0.041, *r* = 0.6 and for T2 vs. T3 *W* = 7 adjusted *p* = 0.036, *r* = 0.61). As expected, there was no effect for the control group (Friedman’s *x*^2^(2) = 2, *p* = 0.3). In sum, the within-group comparisons showed a gradual improvement across testing times for the language-first group. The improvement of the cognitive-first group was large for both post-treatment testing times.

## 4. Discussion

This study was set up to investigate the efficiency of two cross-domain training programs for children with DLD, one linguistic/meta-linguistic and one cognitive. The novelty of this study lies in the alteration of two different training programs which are evaluated in terms of (i) direct, near-, and far-transfer effects on the performance of vSTM and vWM tasks and (ii) the therapy type order, specifically, whether applying linguistic/meta-linguistic training first or applying cognitive training first is more beneficial for vSTM and vWM skills of children with DLD. The particular design of the present study was strongly motivated by the simultaneous presence of linguistic and vSTM/vWM impairments in children with DLD (see Section 1). While the bulk of research concerns the application of linguistic and cognitive methods separately in individuals with DLD, the combination of them has not received any specific attention, although the individuals with DLD might benefit from this combination as they suffer from linguistic and cognitive deficits. A further question is whether the applied therapy type could better trigger the individual response to therapy when delivered first.

With respect to vSTM, the results collectively indicated that non-word repetition improved for both treatment groups. The between-group comparisons for T2 showed that the cognitive-first group was significantly better than the language-first group. This finding can be interpreted as showing that receiving therapy targeting in the specific domain, i.e., cognitive therapy for improving vSTM, maximizes the treatment gains. However, in T3 the language-first group improved significantly compared to T2, while the performance of the cognitive-first group was not increased after receiving the meta-linguistic intervention. This finding can be interpreted as indicative of the therapy type order effect, as it shows that the group that received the meta-linguistic intervention benefited significantly. It may be the case that the meta-linguistic therapy program contributes to the enhancement of vSTM by training the ability to keep and store linguistic information as well as to perform combinational linguistic procedures on it. In our view, these results suggest that far-transfer effects can actually occur for children with DLD when cognitive training is combined with a linguistic training and, more specifically, when a meta-syntactic training is followed by a vWM/vSTM training. These findings corroborate findings from other studies that show far-transfer gains after linguistic training [70,71] and/or vWM/vSTM training [34,35,41,72,73,74]. What our results add to these findings is that the intervention’s positive impact is affected by applying two different therapy types with the linguistic therapy to come first. By contrast, while a significant improvement was observed in the performance of the treatment groups on the forward digit span task, the overall effect of the therapy type order is hard to evaluate as no substantial change occurred between T2 and T3 for the treatment groups. In addition, the improvement of the control group from T1 to T3 indicates that the daily school routine has a positive impact on the vSTM abilities of these children.

With respect to vWM, the results collectively indicated improvement for both the treatment groups especially in T3. While the language-first group showed a gradual improvement that became significant in T3 (as shown by pairwise comparisons), the cognitive-first group showed significant improvement between T1 and T2 as well as between T2 and T3 (with large effects). These results indicate that, with respect to this particular task (BWDS), starting with cognitive therapy can effectively trigger the improvement of vWM abilities. Interestingly, the cognitive-first group starts with lower (although not significantly lower) vWM abilities in this task and shows the same performance as the language-first group in T3. Overall, these findings indicate that receiving therapy targeting a specific domain, i.e., cognitive therapy for improving vWM, positively impacts on the vWM performance as shown by the significance of the difference between T1 and T2 for the cognitive-first group.

Notably, in T3, when both treatment groups had received both therapies, the treatment groups were significantly better than the control and the effect was large. This finding indicates that applying more than one therapy type may maximize the beneficial effect. In fact, despite differences in the improvement rate (gradual improvement across testing times for the language-first group; large improvement for the cognitive-first group) at the end both the treatment groups greatly benefited.

Comparing the within-group performance over different times for the vSTM task (non-word repetition) and the vWM task, a reverse pattern is shown regarding the impact that the therapy type order has on the treatment groups’ performance. Specifically, while the language-first group benefited more from receiving the language therapy first regarding vSTM, the cognitive-first group benefited more from receiving the cognitive therapy first regarding the vWM. Apparently, performance on vSTM tasks may be more closely related to linguistic demands than that on vWM. While current research indicates correlation between vSTM, vWM, and linguistic abilities [36,41,51,52], our results indicate that vSTM performance is more positively affected than vWM performance by linguistic training.

While direct comparison of the outcomes of the present mixed treatment intervention cannot be performed, as to the best of our knowledge a meta-syntactic and cognitive treatment was not simultaneously applied, it should be pointed out that beneficial effects of mixed therapy have been previously reported. In particular, Tyler et al. [74] investigated the effectiveness of a mixed therapeutic design targeting phonology and morphosyntax in children with deficient phonological and morphosyntactic abilities. They found that both treatment approaches were effective. However, they noticed that when the morphosyntactic therapy was delivered, there was a slightly better morphosyntactic improvement. While results of the present study cannot be compared directly with the results by Tyler et al., it is pointed out that the mixed therapeutic designs can reveal aspects of improvement that cannot be shown by the application of one only intervention method.

Overall, the results of this study indicate that far-transfer effects can actually occur for children with DLD (language therapy can affect vSTM and vWM) in addition to direct and near-transfer effects. Furthermore, these data show that the combination of different treatment methods and especially the treatment type order may be a significant matter in improving deficient abilities in DLD. However, this study has the following limitations. First, the combination of different methods has not been compared to the application of one only treatment method (linguistic or cognitive). Second, no assessment of the long-term therapy effects has been performed, as this study was designed to assess the short-term treatment benefits. Therefore, future studies should address the contribution of different methods in comparison to the effectiveness of one only treatment method (linguistic or cognitive) to provide more accurate assessment of the method combination outcome. In addition, the long-term therapy gains should be evaluated by providing assessment a long time after therapy completion.

## 5. Conclusions

Concluding, the present study provides insights into the effects of different therapy methods applied in different orders to individuals with a primary disorder in language. It evaluates their effectiveness simultaneously and assesses short-term gains. By so doing, it shows perspectives on the combination of therapy protocols and the consideration of ‘what comes first’ in the treatment application. There are limitations in the present study that concern the comparison of the combined methods’ effects to the effects of one treatment method; in addition, no long-term assessment was provided after therapy completion. Future studies are expected to address these questions by extending the present therapy protocol to provide further comparisons of different treatment methods and assess long-term benefits, which are crucial for contributing to the well-being of individuals with language disorders.

## Figures and Tables

**Table 1:** Demographic characteristics of the three groups and their performance on non-verbal IQ test (WISC-III) [56] as well as in syntactic comprehension [15].

	Group A (Ν = 16)	Group B (Ν = 17)	Group C (Ν = 16)	Statistics for the Comparisons among Groups
	Μ (SD)	Μ (SD)	Μ (SD)	
Age (in months)	114.56 (18.10)	122.06 (12.40)	112.81 (15.80)	*x*^2^(2) = 2.7, *p* = 0.3
Non-verbal IQ	96.19 (10.55)	100.06 (9.84)	97.88 (11.19)	*x*^2^(2) = 1.1, *p* = 0.6
Syntactic comprehension (accuracy %)	62.50 (13.42)	57.68 (11.43)	61.81 (11.72)	*F*(2, 46) = 0.76, *p* = 0.47
Boys/girls	10/6	11/6	12/4	

**Table 2:** Performance of groups A, B, and C in the NWR, Forward and Backward digit span tasks, in each of the three assessments: pre-training (T1), 1st post-training (T2), 2nd post-training (T3).

	T1 M (SD)	T2 M (SD)	T3 M (SD)
*NWR*			
Group A	0.45 (0.12)	0.56 (0.10)	0.74 (0.05)
Group B	0.48 (0.07)	0.66 (0.05)	0.68 (0.07)
Group C	0.53 (0.13)	0.51 (0.11)	0.52 (0.11)
*Forward digit span*			
Group A	7.38 (1.54)	9.69 (1.82)	10.44 (1.09)
Group B	8.00 (1.12)	9.76 (1.56)	10.12 (1.93)
Group C	8.06 (1.61)	8.44 (1.50)	8.81 (1.11)
*Backward digit span*			
Group A	3.00 (0.82)	4.13 (1.26)	4.88 (1.20)
Group B	2.71 (1.45)	3.76 (1.35)	4.76 (1.39)
Group C	2.69 (0.70)	3.13 (1.15)	2.88 (1.02)

**Table 3:** Contrasts, coefficients (*β*), *t*-values, and *p*-values as well as effect size for each interaction concerning the performance in the Non-Word Repetition task.

Contrast	*β*	*t*(df)	*p*-Value	Effect Size, *r*
Group 2-1: Time 2-1: Comparison between language first and cognitive first concerning the change between testing time 1 and testing time 2	6	2 (92)	0.03	0.2 small
Group 3-2: Time 2-1: Comparison between cognitive first and controls concerning the change between testing time 1 and testing time 2	−20	−7 (92)	0.0000	0.59 large
Group 2-1: Time 3-2: Comparison between language first and cognitive first concerning the change between testing time 2 and testing time 3	−16	−6 (92)	0.0000	0.53 large
Group 3-2: Time 3-2: Comparison between cognitive first and controls concerning the change between testing time 2 and testing time 3	−3	1.1 (92)	0.36	0.09 very small

## Data Availability

The data presented in this study are available upon request from the corresponding author. The data are not publicly available due to ethical reasons.

## References

[1] Bishop D.V.M., Snowling M.J., Thompson P.A., Greenhalgh T., CATALISE-2 Consortium (2017). Phase 2 of CATALISE: A multinational and multidisciplinary Delphi consensus study of problems with language development: Terminology. J. Child Psychol. Psychiatry.

[2] Norbury C.F., Gooch D., Wray C., Baird G., Charman T., Simonoff E., Vamvakas G., Pickles A. (2016). The impact of nonverbal ability on prevalence and clinical presentation of language disorder: Evidence from a population study. J. Child Psychol. Psychiatry.

[3] Bishop D.V.M., Snowling M.J., Thompson P.A., Greenhalgh T., CATALISE Consortium (2016). CATALISE: A multinational and multidisciplinary Delphi consensus study: Identifying Language Impairments in Children. PLoS ONE.

[4] Fey M.E., Catts H.W., Proctor-Williams K., Tomblin J.B., Zhang X. (2004). Oral and Written Story Composition Skills of Children with Language Impairment. J. Speech Lang. Hear. Res..

[5] Georgiou G.P., Theodorou E. (2023). Abilities of children with developmental language disorders in perceiving phonological, grammatical, and semantic structures. J. Autism Dev. Disord..

[6] Leonard L.B. (2014). Children with Specific Language Impairment.

[7] Montgomery J.W., Gillam R.B., Evans J.L., Sergeev A.V. (2017). “Whatdunit?” Sentence Comprehension Abilities of Children with SLI: Sensitivity to Word Order in Canonical and Noncanonical Structures. J. Speech Lang. Hear. Res..

[8] Arosio F., Silleresi S., Guasti M.T. (2024). The production of relative clauses in Italian-speaking children with DLD. Isogloss. Open J. Roman. Linguist..

[9] Fonteneau E., Van Der Lely H.K.J. (2008). Electrical Brain Responses in Language-Impaired Children Reveal Grammar-Specific Deficits. PLoS ONE.

[10] Friedmann N., Novogrodsky R. (2011). Which questions are most difficult to understand?. Lingua.

[11] Hesketh A. (2006). The use of relative clauses by children with language impairment. Clin. Linguist. Phon..

[12] Hestvik A., Schwartz R.G., Tornyova L. (2010). Relative Clause Gap-Filling in Children with Specific Language Impairment. J. Psycholinguist. Res..

[13] Stavrakaki S. (2001). Comprehension of Reversible Relative Clauses in Specifically Language Impaired and Normally Developing Greek Children. Brain Lang..

[14] Stavrakaki S., Fava E. (2002). A-bar Movement Constructions in Greek Children with SLI. Clinical Linguistics: Theory and Applications in Speech Pathology and Therapy.

[15] Talli I., Stavrakaki S. (2020). Short-term memory, working memory and linguistic abilities in bilingual children with Developmental Language Disorder. First Lang..

[16] Graf Estes K., Evans J.L., Else-Quest N.M. (2007). Differences in the Nonword Repetition Performance of Children with and Without Specific Language Impairment: A Meta-Analysis. J. Speech Lang. Hear. Res..

[17] Vugs B., Hendriks M., Cuperus J., Knoors H., Verhoeven L. (2017). Developmental Associations Between Working Memory and Language in Children with Specific Language Impairment: A Longitudinal Study. J. Speech Lang. Hear. Res..

[18] Archibald L.M.D., Joanisse M.F. (2009). On the Sensitivity and Specificity of Nonword Repetition and Sentence Recall to Language and Memory Impairments in Children. J. Speech Lang. Hear. Res..

[19] Ebert K.D., Kohnert K. (2011). Sustained Attention in Children with Primary Language Impairment: A Meta-Analysis. J. Speech Lang. Hear. Res..

[20] Im-Bolter N., Johnson J., Pascual-Leone J. (2006). Processing Limitations in Children with Specific Language Impairment: The Role of Executive Function. Child Dev..

[21] Montgomery J.W., Magimairaj B.M., Finney M.C. (2010). Working Memory and Specific Language Impairment: An Update on the Relation and Perspectives on Assessment and Treatment. Am. J. Speech Lang. Pathol..

[22] Pauls L.J., Archibald L.M.D. (2016). Executive Functions in Children with Specific Language Impairment: A Meta-Analysis. J. Speech Lang. Hear. Res..

[23] Ullman M.T., Pierpont E.I. (2005). Specific Language Impairment is not Specific to Language: The Procedural Deficit Hypothesis. Cortex.

[24] Rice M.L., Wexler K., Marquis J., Hershberger S. (2000). Acquisition of Irregular Past Tense by Children with Specific Language Impairment. J. Speech Lang. Hear. Res..

[25] Novogrodsky R., Friedmann N. (2006). The production of relative clauses in syntactic SLI: A window to the nature of the impairment. Adv. Speech Lang. Pathol..

[26] Stavrakaki S. (2006). Developmental perspectives on Specific Language Impairment: Evidence from the production of wh-questions by Greek SLI children over time. Adv. Speech Lang. Pathol..

[27] Van Der Lely H.K.J., Jones M., Marshall C.R. (2011). Who did Buzz see someone? Grammaticality judgement of wh-questions in typically developing children and children with Grammatical-SLI. Lingua.

[28] Archibald L.M.D., Gathercole S.E. (2006). Short-term and working memory in specific language impairment. Int. J. Lang. Commun. Disord..

[29] Plym J., Lahti-Nuuttila P., Smolander S., Arkkila E., Laasonen M. (2021). Structure of Cognitive Functions in Monolingual Preschool Children with Typical Development and Children with Developmental Language Disorder. J. Speech Lang. Hear. Res..

[30] Gillam R.B., Montgomery J.W., Evans J.L., Gillam S.L. (2019). Cognitive predictors of sentence comprehension in children with and without developmental language disorder: Implications for assessment and treatment. Int. J. Speech Lang. Pathol..

[31] Balthazar C.H., Ebbels S., Zwitserlood R. (2020). Explicit Grammatical Intervention for Developmental Language Disorder: Three Approaches. Lang. Speech Hear. Serv. Sch..

[32] Ebbels S. (2007). Teaching grammar to school-aged children with specific language impairment using Shape Coding. Child Lang. Teach. Ther..

[33] Ebbels S., Norbury C.F., Tomblin J.B., Bishop D.V.M. (2008). Improving grammatical skill in children with specific language impairment. Understanding Developmental Language Disorders: From Theory to Practice.

[34] Stanford E., Durrleman S., Delage H. (2019). The Effect of Working Memory Training on a Clinical Marker of French-Speaking Children with Developmental Language Disorder. Am. J. Speech Lang. Pathol..

[35] Delage H., Stanford E., Durrleman S. (2021). Working memory training enhances complex syntax in children with Developmental Language Disorder. Appl. Psycholinguist..

[36] Pauls L.J., Archibald L.M. (2021). Cognitive and linguistic effects of narrative-based language intervention in children with Developmental Language Disorder. Autism Dev. Lang. Impair..

[37] Alloway T.P., Bibile V., Lau G. (2013). Computerized working memory training: Can it lead to gains in cognitive skills in students?. Comput. Hum. Behav..

[38] Dunning D.L., Holmes J., Gathercole S.E. (2013). Does working memory training lead to generalized improvements in children with low working memory? A randomized controlled trial. Dev. Sci..

[39] Holmes J., Gathercole S.E. (2014). Taking working memory training from the laboratory into schools. Educ. Psychol..

[40] Wener S.E., Archibald L.M. (2011). Domain-specific treatment effects in children with language and/or working memory impairments: A pilot study. Child Lang. Teach. Ther..

[41] Pauls L.J., Archibald L.M.D. (2022). Cognitive and Linguistic Effects of Working Memory Training in Children with Corresponding Deficits. Front. Educ..

[42] Klingberg T., Fernell E., Olesen P.J., Johnson M., Gustafsson P., Dahlström K., Gillberg C.G., Forssberg H., Westerberg H. (2005). Computerized Training of Working Memory in Children with ADHD—A Randomized, Controlled Trial. J. Am. Acad. Child Adolesc. Psychiatry.

[43] Henry L.A., Christopher E., Chiat S., Messer D.J. (2022). A Short and Engaging Adaptive Working-Memory Intervention for Children with Developmental Language Disorder: Effects on Language and Working Memory. Brain Sci..

[44] Cleave P.L., Becker S.D., Curran M.K., Van Horne A.J.O., Fey M.E. (2015). The Efficacy of Recasts in Language Intervention: A Systematic Review and Meta-Analysis. Am. J. Speech Lang. Pathol..

[45] Cirrin F.M., Gillam R.B. (2008). Language Intervention Practices for School-Age Children with Spoken Language Disorders: A Systematic Review. Lang. Speech Hear. Serv. Sch..

[46] Law J., Garrett Z., Nye C. (2004). The Efficacy of Treatment for Children with Developmental Speech and Language Delay/Disorder: A Meta-Analysis. J. Speech Lang. Hear. Res..

[47] Rinaldi S., Caselli M.C., Cofelice V., D’amico S., De Cagno A.G., Della Corte G., Di Martino M.V., Di Costanzo B., Levorato M.C., Penge R. (2021). Efficacy of the Treatment of Developmental Language Disorder: A Systematic Review. Brain Sci..

[48] Storkel H.L., Komesidou R., Fleming K.K., Romine R.S. (2017). Interactive Book Reading to Accelerate Word Learning by Kindergarten Children with Specific Language Impairment: Identifying Adequate Progress and Successful Learning Patterns. Lang. Speech Hear. Serv. Sch..

[49] Nair V.K., Clark G.T., Siyambalapitiya S., Reuterskiöld C. (2023). Language intervention in bilingual children with developmental language disorder: A systematic review. Int. J. Lang. Commun. Disord..

[50] Bettelli G., Guasti M.T., Ajmone P.F., Tenca E., Arosio F. (2023). The effect of a priming-based training on the production of object clitic pronouns in Italian speaking children with DLD. Clin. Linguist. Phon..

[51] Park J., Ritter M., Lombardino L.J., Wiseheart R., Sherman S. (2013). Phonological awareness intervention for verbal working memory skills in school-age children with specific language impairment and concomitant word reading difficulties. Int. J. Res. Stud. Lang. Learn..

[52] Van Kleeck A., Gillam R.B., Hoffman L.M. (2006). Training in phonological awareness generalizes to phonological working memory: A preliminary investigation. J. Speech Lang. Pathol. Appl. Behav. Anal..

[53] Swanson L.A., Fey M.E., Mills C.E., Hood L.S. (2005). Use of Narrative-Based Language Intervention with Children Who Have Specific Language Impairment. Am. J. Speech Lang. Pathol..

[54] Shahmahmood Toktam M., Zahra S., AliPasha M., Ali M., Shahin N. (2018). Cognitive and language intervention in primary language impairment: Studying the effectiveness of working memory training and direct language intervention on expansion of grammar and working memory capacities. Child Lang. Teach. Ther..

[55] Pauls L. (2018). Exploring Associations between Language and Working Memory Abilities in Children with Specific or Combined Impairments in Language and Working Memory. Ph.D. Thesis.

[56] Georgas J., Paraskevopoulos I., Bezevegis I., Giannitsas N. (1997). WISC-III GR: Wechsler Intelligence Scale for Children.

[57] Padeliadu S., Antoniou F. (2007). Reading Test (Test-A).

[58] Talli I. (2010). Linguistic Abilities in Developmental Dyslexia and Specific Language Impairment: A Comparative and Cross-Linguistic Approach. Ph.D. Thesis.

[59] Talli I., Kotsoni P., Stavrakaki S., Sprenger-Charolles L. (2023). Assessing phonological short-term memory in Greek: Reliability and validity of a non-word repetition test. Front. Psychol..

[60] Sprenger-Charolles L., Colé P., Béchennec D., Kipffer-Piquard A. (2005). French normative data on reading and related skills from EVALEC, a new computerized battery of tests (end Grade 1, Grade 2, Grade 3, and Grade 4). Eur. Rev. Appl. Psychol..

[61] Bachourou T. (2021). Γνωστικές Και Γλωσσικές Μέθοδοι Κλινικής Παρέμβασης Στην Ειδική Γλωσσική Διαταραχή. Μια Συγκριτική Μελέτη Της Aποτελεσματικότητάς Τους. (Cognitive and Language Methods of Clinical Intervention in Special Language Impairment: A Comparative Study of Their Efficiency). Ph.D. Thesis.

[62] Delage H., Stanford E., Baratti C., Durrleman S. (2023). Working memory training in children with developmental language disorder: Effects on complex syntax in narratives. Front. Rehabil. Sci..

[63] Bachourou T., Bachourou A. (2015). Assisted Language Acquisition (AS.L.A.). Software. https://lab7.gr/asla.html.

[64] Bryan A., Chiat S., Marshall J. (1997). Colourful semantics: Thematic role therapy. Language Disorders in Children and Adults.

[65] Spooner L. (2002). Addressing expressive language disorder in children who also have severe receptive language disorder: A psycholinguistic approach. Child Lang. Teach. Ther..

[66] Bachourou T., Bachourou A. (2015). BOOM! (Boosting Memory). Software. https://lab7.gr/boom.html.

[67] Salis C., Kelly H., Code C. (2015). Assessment and treatment of short-term and working memory impairments in stroke aphasia: A practical tutorial: Memory and aphasia. Int. J. Lang. Commun. Disord..

[68] Salis C. (2012). Short-term memory treatment: Patterns of learning and generalisation to sentence comprehension in a person with aphasia. Neuropsychol. Rehabil..

[69] R Core Team (2023). R: A Language and Environment for Statistical Computing. https://www.R-project.org/.

[70] Gillam R.B., Loeb D.F., Hoffman L.M., Bohman T., Champlin C.A., Thibodeau L., Widen J., Brandel J., Friel-Patti S. (2008). The Efficacy of Fast ForWord Language Intervention in School-Age Children with Language Impairment: A Randomized Controlled Trial. J. Speech Lang. Hear. Res..

[71] Stevens C., Fanning J., Coch D., Sanders L., Neville H. (2008). Neural mechanisms of selective auditory attention are enhanced by computerized training: Electrophysiological evidence from language-impaired and typically developing children. Brain Res..

[72] Ebert K.D., Kohnert K., Pham G., Rentmeester Disher J., Payesteh B. (2014). Three Treatments for Bilingual Children with Primary Language Impairment: Examining Cross-Linguistic and Cross-Domain Effects. J. Speech Lang. Hear. Res..

[73] Ebert K.D., Rentmeester-Disher J., Kohnert K. (2012). Nonlinguistic cognitive treatment for bilingual children with primary language impairment. Clin. Linguist. Phon..

[74] Tyler A.A., Lewis K.E., Haskill A., Tolbert L.C. (2002). Efficacy and cross-domain effects of a morphosyntax and a phonology intervention. Lang. Speech Hear. Ser. Sch..

